# Enrichment experiment changes microbial interactions in an ultra-oligotrophic environment

**DOI:** 10.3389/fmicb.2015.00246

**Published:** 2015-04-01

**Authors:** Gabriel Y. Ponce-Soto, Eneas Aguirre-von-Wobeser, Luis E. Eguiarte, James J. Elser, Zarraz M.-P. Lee, Valeria Souza

**Affiliations:** ^1^Laboratorio de Ecología Molecular y Experimental, Departamento de Ecología Evolutiva, Instituto de Ecología, Universidad Nacional Autónoma de MéxicoCoyoacán, México; ^2^Red de Estudios Moleculares Avanzados, Instituto de Ecología A.C.Xalapa, México; ^3^School of Life Sciences, Arizona State UniversityTempe, AZ, USA

**Keywords:** mesocosm, nutrient enrichment, Cuatro Cienegas Basin, proteobacteria interactions, community structure

## Abstract

The increase of nutrients in water bodies, in particular nitrogen (N) and phosphorus (P) due to the recent expansion of agricultural and other human activities is accelerating environmental degradation of these water bodies, elevating the risk of eutrophication and reducing biodiversity. To evaluate the ecological effects of the influx of nutrients in an oligotrophic and stoichiometrically imbalanced environment, we performed a replicated *in situ* mesocosm experiment. We analyzed the effects of a N- and P-enrichment on the bacterial interspecific interactions in an experiment conducted in the Cuatro Cienegas Basin (CCB) in Mexico. This is a desert ecosystem comprised of several aquatic systems with a large number of microbial endemic species. The abundance of key nutrients in this basin exhibits strong stoichiometric imbalance (high N:P ratios), suggesting that species diversity is maintained mostly by competition for resources. We focused on the biofilm formation and antibiotic resistance of 960 strains of cultivated bacteria in two habitats, water and sediment, before and after 3 weeks of fertilization. The water habitat was dominated by *Pseudomonas*, while *Halomonas* dominated the sediment. Strong antibiotic resistance was found among the isolates at time zero in the nutrient-poor bacterial communities, but resistance declined in the bacteria isolated in the nutrient-rich environments, suggesting that in the nutrient-poor original environment, negative inter-specific interactions were important, while in the nutrient-rich environments, competitive interactions are not so important. In water, a significant increase in the percentage of biofilm-forming strains was observed for all treatments involving nutrient addition.

## Introduction

A central goal of ecology is the understanding of the driving principles underpinning biodiversity (Gaston, 2000). Several lines of research have tried to explain the differences in diversity among local communities, focusing on approaches ranging from network interactions in food webs to random assemblages resulting from the dispersion capacity of organisms (Hutchinson, 1959; Dykhuizen, 1998; Kassen et al., 2000; Bennie et al., 2006; Marini et al., 2007). These theories, as well as other more complex explanations of biodiversity (such as chaotic interactions between competing strains; Huisman and Weissing, 1999) are partially successful, depending on the system studied and are frequently complementary. The patterns of biodiversity are determined by the combined impacts of interactions of several biotic and abiotic environmental factors, and cost-benefit strategies followed by each species can change according to the complexity of the community and its nutrient availability (Marini et al., 2007; Werner et al., 2014).

For instance, it is known that the abundances and proportions of nitrogen and phosphorus in the environment have a major influence in the composition of species at macro and microscopic scales (Makino and Cotner, 2005; Jansson et al., 2006; Marini et al., 2007; Christofoli et al., 2010; Nelson and Carlson, 2011). Classic trade-off competition theory places resource availability at the center of the processes that influence community structure (Tilman et al., 1981; Smith, 1993; Brauer et al., 2012). MacArthur and Wilson (1967) coined the terms “r-selection” and “K-selection.” This theory takes into account biotic and abiotic factors such as climate, mortality, survivorship, population size, intra and interspecific competition, relative abundance and length of life. Under this theory, r-strategists are adapted to abundant nutrients, which are rapidly exploited. On the other hand, K-strategists are adapted to a long-term survival on limited resources (Pianka, 1970; Fuchs et al., 2000; Singer et al., 2011). Experimental evidence from different microcosm experiments shows that nitrogen and phosphorus enrichments result in significant changes in community structure in terms of uniformity and species richness (Schäfer et al., 2001; Nelson and Carlson, 2011).

The changes in community structure are probably driven mainly by the effects of nutrients on the growth rates of individual strains (Smith, 1993), but other factors could also play a role such as seasonal changes (Rodríguez-Verdugo et al., 2012; Bevivino et al., 2014), as well as changes in mutualistic interactions due to fluctuations in the supply and demand of “public goods” by cause of the entrance of nutrients to the ecosystem (Morris et al., 2012; Sachs and Hollowell, 2012; Werner et al., 2014).

The release of chemical compounds into the environment, which are toxic or inhibitory to the competitors, is one of the commonly observed antagonistic interactions (Riley and Gordon, 1999; Lenski and Riley, 2002; Riley and Wertz, 2002; Kirkup and Riley, 2004; Hibbing et al., 2010; Kohanski et al., 2010; Majeed et al., 2011, 2013; Pérez-Gutiérrez et al., 2013; Aguirre-von-Wobeser et al., 2014). These secretions highly influence community structure and maintain cohesion of bacterial populations by leading to the extinction of sensitive strains in liquid media, but to coexistence in a structured media (Validov et al., 2005; Greig and Travisano, 2008; Rypien et al., 2010; Cordero et al., 2012). In the non-transitive model rock-paper-scissors (RPS), one antagonist, one sensitive and one resistant strain coexist in structured media (Czarán et al., 2002; Kerr et al., 2002; Kirkup and Riley, 2004). In a natural environment, this non-transitive relation may occur if toxic production is costly, both sensitive and resistant strains exist and costs associated with resistance are less than those of toxin production. However, the relative magnitude of each of these features is critical for coexistence (Kerr et al., 2002).

The effects of nutrient addition in microbial community structure can also depend on the extant biodiversity prior to the increase of nutrient availability, as shown by an experiment performed on the bacterial community of Owasso Lake (Minnesota, USA), which has been reported as one of the least diverse bacterial communities known (Makino and Cotner, 2005). When this lake was enriched with nitrogen, phosphorus and carbon, the community showed a response that would be expected from a single strain, rather than from a community (Jürgens and Güde, 1990), suggesting that if bacterial diversity in a given environment is low, this diversity will remain low, because of its low potential to respond at a community level to changes in its environment. In contrast, in more diverse environments, nutrient enrichment usually homogenizes the composition of the community assemblage (Donohue et al., 2009).

The Cuatro Cienegas Basin (CCB) located at the Chihuahuan desert of north central Mexico is a good system to study the effects of the addition of nutrients on bacterial communities. CCB harbors a number of highly oligotrophic aquatic ecosystems that have very low available phosphorus levels, as well as a stoichiometric disequilibrium with nitrogen and thus are strongly limited by phosphorus (Elser et al., 2005, 2006). We have suggested that the high microbial species diversity in CCB is strongly shaped by the stress of low nutrient supplies and the impacts of interspecific competition (Souza et al., 2008). Previous studies indicate that CCB bacterial communities display strategies to cope with this lack of nutrients, including a high representation of genes involved in phosphorus assimilation (e.g., *pho* and *pst* in Mesquites river; Breitbart et al., 2009) and the presence of a large number of genes related to the production and resistance to antibiotics (in Pozas Rojas; Bonilla-Rosso et al., 2012; Peimbert et al., 2012).

*Pseudomonas* and other cultivable proteobacteria are abundant in different aquatic systems in CCB, including the Churince system (Escalante et al., 2009) and in the Los Hundidos region (Bonilla-Rosso et al., 2012; Peimbert et al., 2012). In a different study in the flow system Churince, it was found that the *Pseudomonas* genus exhibited a seasonal variation across summer and winter (Rodríguez-Verdugo et al., 2012).

In this paper, we analyze the seasonal response of bacteria cultivable in *Pseudomonas* Isolation Agar (PIA; Difco^1^, Detroit, MI) in response to the nutrient amendment. Our study seeks to understand the effects of nitrogen and phosphorus fertilization on the interaction potential of the cultivable gamma-proteobacteria community in two different habitats, water and sediment, by determining how nutrient enrichment affected various features related to microbial species interactions, such as the tendency to form biofilms and resistance to antibiotic compounds. We are clearly aware of the limitations of culture media in order to capture the diversity of a site, as we have described elsewhere (see Souza et al., 2006, 2012), nevertheless, only in culture we can study the physiology and sociology of particular strains, which is the particular aim of this study. Our findings shed light on how the supplies of key nutrients, such as N and P modulate community structure, as well as the nature and intensity of interspecific interactions.

## Materials and methods

### Study site

The nutrient enrichment experiment was conducted *in situ* in a small shallow evaporitic pond, Lagunita (26.84810° N, 102.14160° W), lateral to the main Churince flow system. The Churince flow system, located at the western region of CCB is dominated by gypsum sediments and has a strong longitudinal gradient of salinity, temperature, pH and dissolved oxygen (Cerritos et al., 2011). Lagunita is characterized by low phosphorus concentrations (PO_4_ as low as 0.1 μM and often below detection) (Elser et al., 2005), but relatively high concentrations of inorganic N and thus high N:P ratios (>200:1 for total nutrients; Lee et al., in press). Lagunita is subjected to strong evaporation, the greatest water depth at the beginning of the experiment was 32 cm and decreased to as low as 12 cm by the end of the experiment.

### Experimental design of mesocosm

The mesocosm experiment was conducted from May to June 2011. Each mesocosm consisted of a round clear plastic tube with a diameter of 40 cm. The tube had a depth of 20 cm into the sediment and approximately 20 cm above the water surface. The mesocosms were arranged in a randomized complete block with a total of 5 blocks, separated about 2-4 m from each other. Each block consisted of four treatments. A non enriched control treatment, a phosphorus enrichment treatment (P), amended with KH_2_PO_4_ and maintained at 2 day intervals at a final concentration of 1 μM; a nitrogen and phosphorus enrichment (NP), amended as above with KH_2_PO_4_ to 1 μM but also with NH_4_NO_3_, to achieve N:P of 16:1; and a nitrogen and phosphorus enrichment with extra nitrogen (NNP), amended as above with 1 μM KH_2_PO_4_ but also with NH_4_NO_3_ at an N:P ratio = 75:1.

### Methodology for chemical analyses

For the chemical analyses, water was collected in acid-washed 2 L cubitainers. Water samples were filtered through pre-combusted (24 h at 450°C) GF/F filters (Whatman, Piscataway, NJ) for seston elementary analysis and stored at −20°C. To measure total dissolved nutrients, water samples were filtered through 0.2 μm polyethersulfone membrane filters. Samples for dissolved organic carbon (DOC), and total dissolved nitrogen (TDN) were acidified with 12 N HCL to pH < 2 and stored in the dark at room temperature, while the remaining filtrate was frozen for total dissolved phosphorus (TDP) and soluble reactive phosphorus (SRP) analyses.

GF/F with seston were thawed, dried at 60°C and packed into tin disks (Elemental Microanalysis, U.K.) for N analyses with a Perkin Elmer™ PE 2400 CHN Analyzer at the Arizona State University Goldwater Environmental Laboratory (ASU GEL). Another set of dried GF/F filters from the same water samples was used in order to estimate seston P content. These filters were digested in persulfate followed by a colorimetric analysis to determine PO^3−^_4_ (APHA, 2005). TDP concentrations were determined using the colorimetric assay after persulfate digestion as previously described; SRP was measured without the persulfate digestion. DOC and TDN were analyzed using the Shimadzu TOC-VC/TN analyzer at the ASU GEL. Total Phosphorus (TP) concentration was calculated as the sum of the seston and total dissolved pools.

### Sample collection and processing

Water and sediment samples were taken for each one of the four treatments for each experimental block (20 samples total). Initial samples were obtained from surface water and top of sediment prior to the application of the treatments (T0; 14 May 2011) and after 21 days of enrichment (4 June 2011) using sterile BD Falcon vials (BD Biosciences, San Jose, CA). Cultivable bacteria were obtained by plating 100 μl of each water sample or 100 μl of a 1:10 sediment dilution, prepared with 0.9% NaCl solution. Strains were isolated from water and sediment using PIA medium. Strains were incubated on agar plates at room temperature for 2 days at the field, and then were kept at 4°C until isolation in the laboratory. Individual colonies were transferred to new PIA plates and then were grown at 30°C. A total of 960 isolates were obtained.

### DNA extraction and PCR amplification

Phylogenetic identification of the strains was performed using the 16S rRNA gene. For the isolates, DNA extraction was very complicated, and thus several methods were tested (Chen and Kuo, 1993; Aljanabi and Martinez, 1997; Reischl et al., 2000, and DNeasy Blood and Tissue kits, Qiagen, Hilden, Germany). Ultimately, the DNeasy Blood and Tissue kit was the method used, and from the 960 isolates, good quality DNA was obtained for 152 strains. 16S rRNA genes were amplified using universal primers 27F (5′-AGA GTT TGA TCC TGG CTC AG-3′) and 1492R (5′-GGT TAC CTT GTT ACG ACT T-3′) (Lane, 1991) and high fidelity *Phusion* hot start DNA polymerase (Finnzymes, Espoo, Finland). All reactions were carried out in a Techne TC-3000 thermal cycler (Barloworld Scientific, Staffordshire, UK) with the following program: 94°C for 5 min, followed by 30 cycles consisting of 94°C for 1 min, 50°C for 30 s, 72°C for 1 min and 72°C for 5 min. Polymerase chain reaction (PCR) amplification products were electrophoresed on 1% agarose gels. Sanger sequencing was performed at the University of Washington High Throughput Genomics Center. The sequences have been uploaded to GenBank with accession numbers (KF317734-KF317770, KM352505-KM352636).

### Phylogenetic analysis

The 16S rRNA sequences were aligned with CLUSTALW (Larkin et al., 2007) and MUSCLE (Edgar, 2004), and the alignments were manually revised. For the reconstruction of the phylogenetic tree, a maximum likelihood analysis was done with PhyML version 3.0 (Guindon et al., 2010) with the TrN+G model. The substitution model was calculated with jModelTest 2.1.3 (Darriba et al., 2012). The degree of statistical support for the branches was determined with 1000 bootstrap replicates. Genera level identification of the strains was made using the classifier tool (Wang et al., 2007) from the Ribosomal Database Project (RDP) Release 10, update 30 (Cole et al., 2009; Table 1). We performed a local BLAST search (Altschul et al., 1990) to find the nearest neighbors using the 16S ribosomal RNA. These analyses were performed with 700 bp from the 5′ end of all the sequences.

**Table 1:** **Summary of nutrient-induced shifts in relative abundance of dominant bacterial lineages**.

**Class**	**Consensus clade**	**Total Count**	**T0**		**Control**		**NP**		**NNP**		**P**	
			**H_2_0**	**Sed**	**H_2_0**	**Sed**	**H_2_0**	**Sed**	**H_2_0**	**Sed**	**H_2_0**	**Sed**
α-Proteobacteria	*Rhizobium*	**8**	0	**6**	0	0	0	0	0	0	0	**2**
γ-Proteobacteria	*Stenotrophomonas*	**9**	0	**9**	0	0	0	0	0	0	0	0
γ-Proteobacteria	*Pseudomonas*	**54**	**20**	0	**9**	0	0	0	**4**	0	**21**	0
γ-Proteobacteria	*Shewanella*	**1**	**1**	0	0	0	0	0	0	0	0	0
γ-Proteobacteria	*Aeromonas*	**20**	**5**	0	0	**3**	**10**	0	**2**	0	0	0
γ-Proteobacteria	*Citrobacter*	**1**	**1**	0	0	0	0	0	0	0	0	0
γ-Proteobacteria	*Rheinheimera*	**5**	**5**	0	0	0	0	0	0	0	0	0
γ-Proteobacteria	*Halomonas*	**47**	**7**	0	0	0	0	**18**	0	**22**	0	0
Actinobacteria	*Kocuria*	**4**	0	0	0	0	0	**1**	0	0	0	**3**
Actinobacteria	*Brachybacterium*	**3**	0	**3**	0	0	0	0	0	0	0	0

### Antibiotic resistance assays

The 960 isolates were grown on LB plates supplemented with the following antibiotic concentrations: Carbenicillin 500 μg/ml, Kanamycin, 200 μg/ml, Tetracycline, 150 μg/ml, Streptomycin, 200 μg/ml and Gentamicin 150 μg/ml. Isolates were labeled as resistant to an antibiotic if we observed bacterial growth; otherwise, they were considered as nonresistant.

For statistical inference, the antibiotics data were organized as contingency tables, and Barnard's exact test (Barnard, 1945) was performed. Independent tests were conducted for each treatment against the initial time, for water and sediment data. Since several treatments were compared to the initial condition, a Bonferroni correction was applied for multiple tests.

### Biofilm formation assay

A microtiter dish assay was performed for the same 960 isolates as described by O'Toole (2011) with the following modifications. The overnight culture was grown in LB medium. The microtiter plates were incubated for 18 h at 30°C. Each strain was analyzed by triplicate and we used the strains *P. aeruginosa* PAO1 and *E. coli* MC4100 as positive and negative controls, respectively. After incubation, plates were gently washed with water, and subsequently 125 μl of a 0.1% solution of crystal violet in water was added to each well. The plates were incubated at room temperature for 15 min and then washed with water. 125 μl of 30% acetic acid in water was added to each well to solubilize the crystal violet and it was incubated for 15 min. The volume was transferred to a new flat-bottomed microtiter dish. Absorbance was read with a Synergy HT plate reader (BioTek, Winooski, VT) at 550 nm.

To determine the statistical significance of differences in biofilm formation between the different treatments and the initial condition, we organized the data as contingency tables including the number of positive and negative strains for biofilm formation for the initial time and each treatment, and applied Barnard's exact tests. To correct multiple testing, a Bonferroni correction was applied.

## Results

To characterize the changes in diversity of cultivated Proteobacteria related to pseudomonads associated with nutrient enrichment in a water system, we performed an *in situ* mesocosm experiment with three experimental manipulations, adding phosphate (P), phosphate and nitrogen (NP), and phosphate and excess nitrogen (NNP), as well as an un-enriched control. The experiment is part of a bigger project that is described in detail elsewhere (Lee et al., in press). Cultures were obtained from surface water and sediment in two sampling events, prior to the experimental manipulations, and after 21 days, and a total of 960 isolates were analyzed for interaction phenotypes such as biofilm formation, and antibiotic resistance. A subsample of the isolates was characterized by 16S rRNA sequence.

### Nutrient concentrations

Nutrient conditions during the experiment were characterized by low concentrations of P but relatively high concentrations of N that increased in NP and NNP treatments. Despite nutrient enrichment, N:P ratios remained quite stoichiometrically imbalanced. Except for the NNP treatment, total dissolved phosphorus and soluble reactive phosphorus were significantly different respect to control. Total phosphorus, total dissolved phosphorus and the N:P ratio were significantly different in all enriched treatments. Total dissolved nitrogen was significantly different only in the nitrogen amended treatments (Table 2). Details of dynamics and fate of N and P in the mesocosms are given elsewhere (Lee et al., in press).

**Table 2:** **Nutrient concentrations**.

**Treatment**	**DOC**	**TDN**	**Seston P**	**TP**	**TDP**	**SRP**	**N:P**
T0	2177 ± 51.84	130 ± 5.98	1.21 ± 0.08	1.79 ± 0.20	0.58 ± 0.17	0.06 ± 0.02	47.26 ± 1.95
Control	2799.17 ± 212.81	182.77 ± 9.51	1.09 ± 0.11	2.47 ± 0.24	1.39 ± 0.25	0.31 ± 0.09	49.12 ± 6.04
NP	3005 ± 227.63	202.48 ± 12.11^*^	3.03 ± 0.26^*^	4.17 ± 0.62^*^	1.15 ± 0.40	0.27 ± 0.12	36.39 ± 6.34^*^
NNP	3198.81 ± 144.87^*^	226.32 ± 25.63^*^	3.29 ± 0.68^*^	4.15 ± 0.71^*^	0.86 ± 0.25^*^	0.19 ± 0.08^*^	36.25 ± 6.20^*^
P	3010.65 ± 227.30	192.05 ± 17.88	2.71 ± 0.33^*^	3.95 ± 0.59^*^	1.24 ± 0.28	0.33 ± 0.10	28.26 ± 3.75^*^

Dissolved organic carbon (DOC), total dissolved nitrogen (TDN), total phosphorus (TP), total dissolved phosphorus (TDP) and soluble reactive phosphorus (SRP) concentrations in the pond's water and in each treatment after the nutrient enrichment. All values are in μM L^−1^ with the exception of N:P which represents the elemental stoichiometry of seston. Each value represents the average and one standard deviation. The “

* *” symbol denotes significant change from the control*.

### Phylogenetic diversity and responses to treatments

The phylogenetic relationships of partial 16S rRNA gene sequences (700 bases) of the 152 strains were determined by queries against the Ribosomal Database Project (Cole et al., 2009) using SeqMatch and Classifier (Wang et al., 2007). The phylogenetic results showed that the strain collection was dominated by Proteobacteria (145 isolates/95.4%), with members from the gamma- (137/90.1%) and alpha- (8/5.3%) subdivisions (Table 1), consistent with the use of the PIA medium for isolation. We also found several Actinobacteria (7/4.6%). As described by King et al. (1954), this medium includes Irgasan®, a broad spectrum antimicrobial substance that is not active against *Pseudomonas* spp. The medium also enhances the formation of pigments by *Pseudomonas* by adding magnesium chloride and potassium sulfate. *Pseudomonas* species were found in all un-enriched water cultures as well as in almost all enriched samples, with the exception of the NP treatment. *Pseudomonas* was the most abundant genus for the water isolates and overall for all samples (35.5% of the isolates). *Halomonas* was the most abundant genus among the sediment strains, representing 30.9% of the isolates.

**Figure 1:**
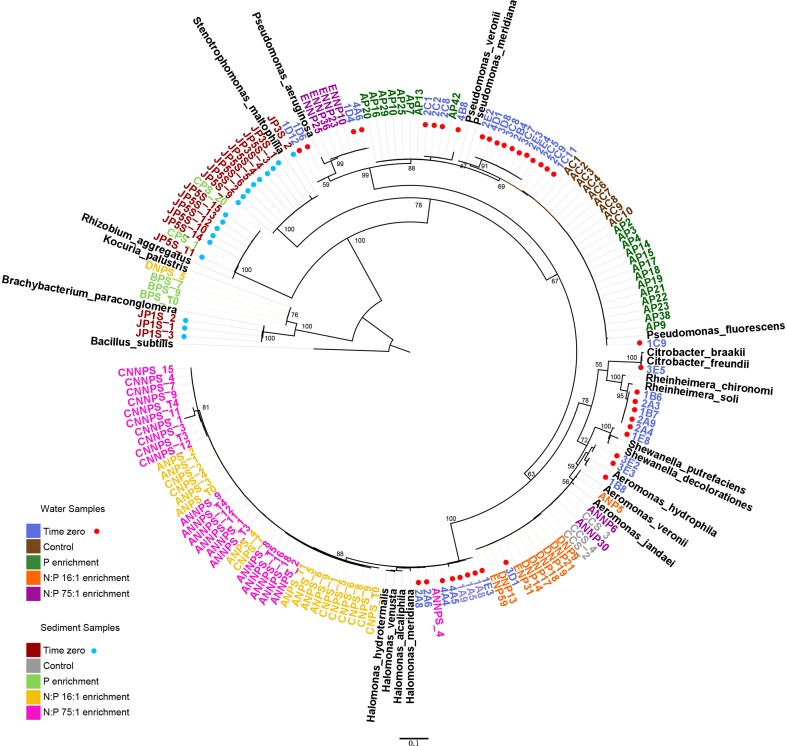
**Phylogenetic relationships among 16S ribosomal RNA genes**. Sequences from the 152 isolates (highlighted in colors) and 21 reference strains. The coded name of the strains corresponds to the treatments and the environment from which they were isolated. Water samples: #[A-E]#, mesocosm T0, XC#, control treatment, XP#, phosphorus enrichment, XNP#, N:P 16:1 treatment, XNNP#, and N:P 75:1 treatment. Sediment samples: JPXs_#, mesocosm T0, XCs_#, control treatment, XPs_#, phosphorus treatment, XNPs_#, N:P 16:1 treatment, XNNPs_#, and N:P 75:1 treatment, where # is a number and X a letter from A to E, each one represents a replicate.

Using the aligned sequences, a maximum likelihood tree was constructed (Figure 1). For any given treatment, groups of closely related, almost identical strains dominated the samples, even though they were obtained from different replicate mesocosms. Previous to the experimental manipulations, only gamma-proteobacteria were retrieved from the water samples, which were dominated by *Pseudomonas*. Other genera were present, including *Aeromonas, Shewanella, Citrobacter, Rheinheimera*, and *Halomonas*. With the addition of phosphorus (P treatment), many closely related *Pseudomonas* were retrieved. In the NP treatment, all the obtained isolates belonged to the genus *Aeromonas*. For the high N:P treatment (NNP), a group of *Pseudomonas* became abundant and two *Aeromonas* isolates were obtained.

Phylogenetic differences were observed between the abundant strains obtained from the different habitats (water vs. sediment) but also between the strains corresponding to the different treatments. At the beginning of the experiment, the sediment samples were dominated by another gamma-proteobacterium, *Stenotrophomonas*, as well as an alpha-proteobacterium, *Rhizobium*. Some Actinobacteria were also isolated from these initial samples, belonging to the genus *Brachybacterium*. With P enrichment, *Rhizobium* was also found, as well as an Actinobacterium, from the genus, *Kocuria*. However, a larger change in the sampled community was found in all treatments including nitrogen along with P (NP and NNP), where *Halomonas* (gamma-proteobacteria) was largely dominant.

### Antibiotic resistance

**Figure 2:**
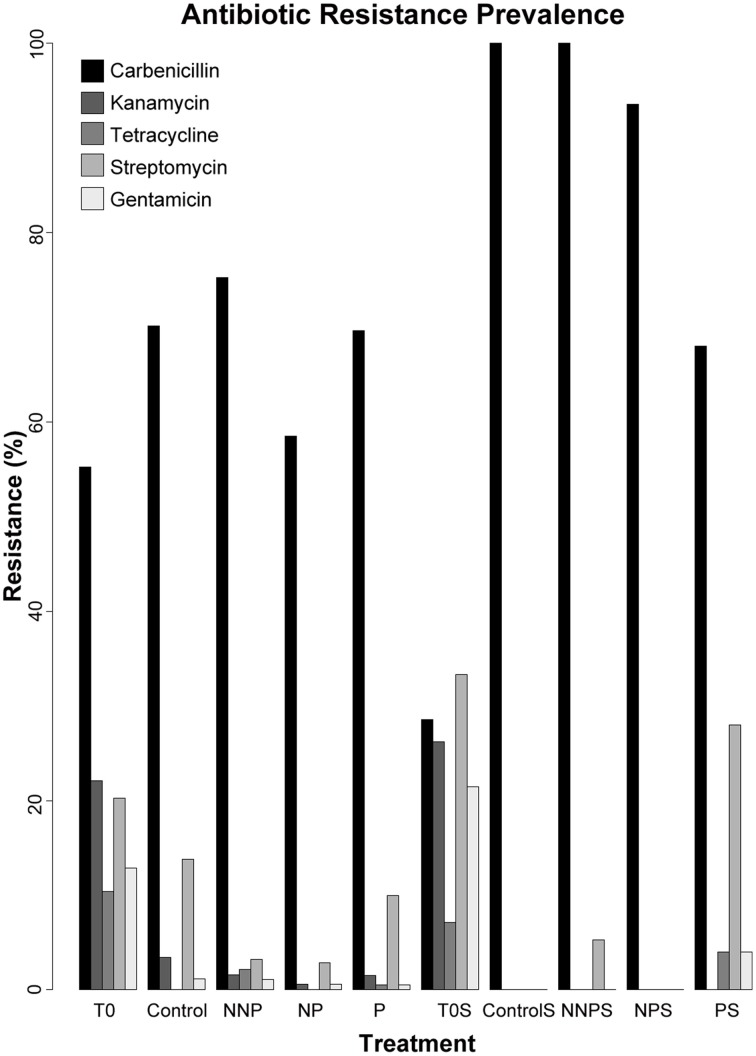
**Prevalence of antibiotic resistance among 960 isolates**. The isolates analyzed for each treatment were as follows. T0—163, Control—87, NNP—186, NP—176, P—201, T0S—42, CS—11, NNPS—38, NPS—31, and PS—25.

We analyzed the prevalence of antibiotic resistances among the 960 isolates. The antibiotics analyzed were Carbenicillin, Kanamycin, Tetracycline, Streptomycin and Gentamicin (Supplementary Table 1; Supplementary Figures 1, 2). Carbenicillin was the most common form of resistance among the isolates. The Carbenicillin resistance was found in 55.2% of the water isolates at time zero and in 68.3% of the total water isolates after the experiment, and in 28.6 and 90.5% of the total sediment isolates, at time zero and after the experiment, respectively. Tetracycline was the least common form of resistance, being represented in 10.4 and 0.8% of the water isolates and 7.1 and 1.0% of the sediment isolates before and after the experiment, respectively. Kanamycin resistance was not observed in sediment samples after the experiment. Overall, there was a statistically significant decrease in the number of resistant strains after the experiment (Bernard's exact test; *p* < 0.05; Figure 2; Table 3), in all three fertilization treatments and in the control, with the marked exception of Carbenicillin, for which resistance increased significantly in NNP and P treatments in water and in all treatments in sediment.

**Table 3:** **Antibiotic resistance**.

**Treatment**	**n**	**Carbenicillin**		**Kanamycin**		**Tetracycline**		**Streptomycin**		**Gentamicin**	
		**Resistant (%)**	***p***	**Resistant (%)**	***P***	**Resistant (%)**	***p***	**Resistant (%)**	***p***	**Resistant (%)**	***p***
Water T0	163	55.21		22.09		10.43		20.25		12.88	
Water Control	87	70.11	0.09	3.45	4.60E-4	0.00	7.23E-3	13.79	0.93	1.15	7.23E-3
Water NNP	186	75.27	3.30E-4	1.61	4.30E-9	2.15	4.94E-3	3.23	1.81E-6	1.08	3.00E-5
Water NP	176	58.52	1.00	0.57	6.04E-10	0.00	4.00E-5	2.84	1.40E-6	0.57	2.00E-5
Water P	201	69.62	0.02	1.49	7.91E-10	0.50	5.00E-5	9.95	0.02	0.50	2.97E-6
Sediment T0	42	28.57		26.19		7.14		33.33		21.43	
Sediment Control	11	100	3.00E-4	0.00	0.38	0.00	1.00	0.00	0.10	0.00	0.38
Sediment NNP	38	100	1.53E-11	0.00	2.54E-3	0.00	0.41	5.26	6.99E-3	0.00	0.01
Sediment NP	31	93.55	4.38E-8	0.00	8.15E-3	0.00	0.63	0.00	1.31E-3	0.00	0.02
Sediment P	25	68.00	6.64E-3	0.00	0.02	4.00	1.00	28.00	1.00	4.00	0.27

*Percentages of strain resistant for different antibiotics, and their significance with respect to the initial condition (T0) in both water and sediment, according to Barnard's exact tests, with Bonferroni correction for multiple tests. Percentages with p-values less than 0.05 are considered significantly different than the corresponding to T0 values*.

### Biofilm formation

**Figure 3:**
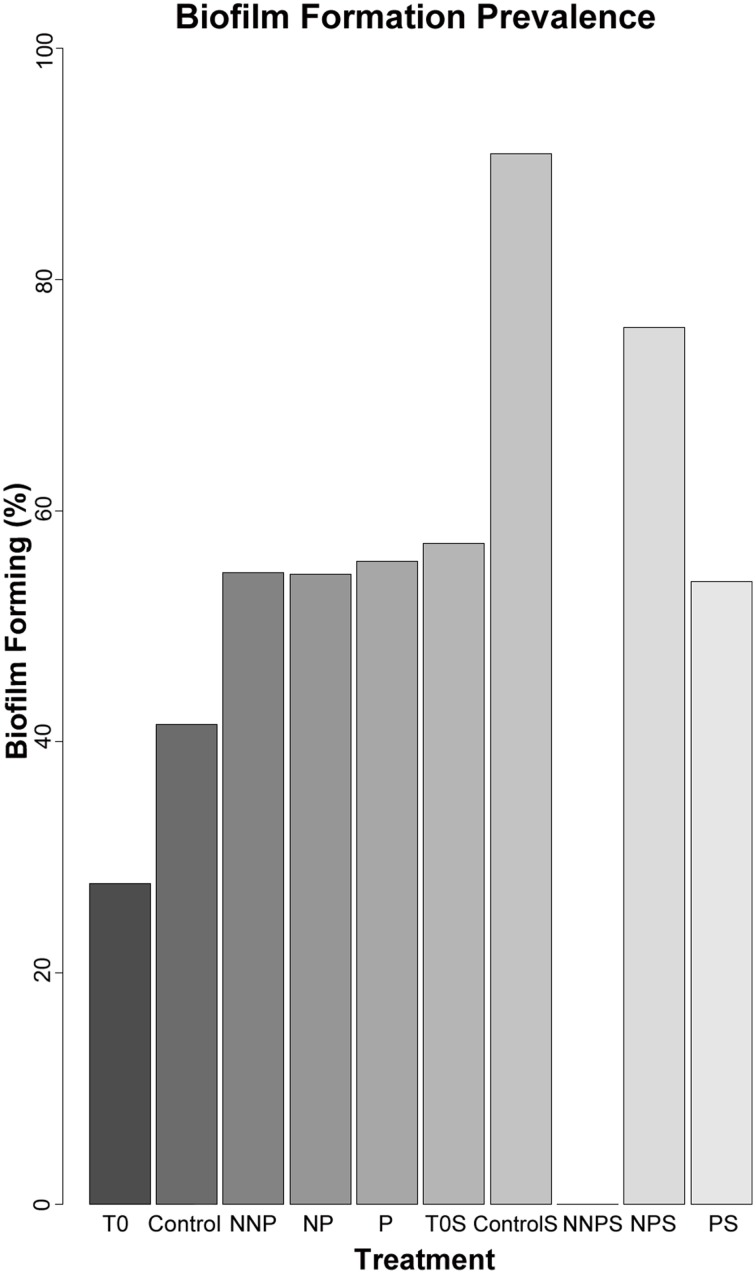
**Relative frequency of biofilm forming strains among 923 isolates**. The isolates analyzed for each treatment were as follows. T0—157, Control—84, NNP—174, NP—167, P—196, T0S—42, CS—11, NNPS—38, NPS—29, and PS—25.

To further characterize the ecological traits of the studied strains, biofilm formation was analyzed for 923 isolates. In the water samples, the enriched isolates exhibited an increase in the proportion of the ability to form biofilm (Figure 3; Supplementary Figure 1). In a structured environment, interactions can be enhanced by biofilm formation. As expected, sediment strains generally had a greater tendency to produce biofilm that those isolated from water. However, in sediment, differences between before and after the treatments were not significant (Barnard's exact test; *p* > 0.05; Table 4), except for the NNP treatment, for which no biofilm-producing strains were detected (Figure 3; Supplementary Figure 2). In the case of water, a significant increase in the percentage of biofilm-producing strains was observed for all treatments involving nutrient additions, namely P, NP, and NNP (Barnard's exact test; *p* < 0.05; Table 4).

**Table 4:** **Biofilm formation**.

**Treatment**	***n***	**Biofilm forming (%)**	***P***
Water T0	155	27.74	
Water Control	82	41.46	0.13
Water NNP	174	54.60	3.07E-6
Water NP	167	54.49	4.37E-6
Water P	196	55.61	5.78E-7
Sediment T0	43	57.14	
Sediment Control	11	90.91	0.16
Sediment NNP	38	0.00	5.82E-8
Sediment NP	31	75.86	0.50
Sediment P	26	53.85	1.00

*Percentages of strain forming biofilm, and their significance with respect to the initial condition (T0) in both water and sediment, according to Barnard's exact tests, with Bonferroni correction for multiple tests. Percentages with p-values less than 0.05 are considered significantly different than the corresponding to T0 values*.

## Discussion

In this study, the effect of experimental nutrient enrichment in a shallow, nutrient-deficient pond was analyzed to assess changes in the composition of the cultivable microbial community as a function of the N:P ratio of enrichment, as well as the modifications in the interaction network among cultivable isolates. The selective medium PIA was found to be highly selective to gamma-proteobacteria, a class previously reported as abundant at Churince as well as other sites at CCB (Souza et al., 2006; Escalante et al., 2009; Bonilla-Rosso et al., 2012) and it has not been analyzed in detail. Unfortunately, a great amount of the isolates could not be identified, as it seems that Cuatro Ciénegas bacteria produce compounds that inhibit the PCR reaction, compounds that remain despite the use of several DNA extraction methods. To analyze the community response to the nutrient enrichment without the cultivation bias, 16S libraries from water and sediment samples, from May to June 2011, were also sequenced. The analysis of the community showed, in agreement with this study, a shift in the community composition with the disappearance of several bacterial genera after the increased nutrient availability (Elser et al., 2014, in preparation).

Our hypothesis that Lagunita is P-limited was supported, as it was found that all added P that remained in the water column was immobilized into seston (Table 2). This is reflected by the fact that TDP and SRP were not significantly different respect to the control, but TP and phosphorus in seston were. This difference was also observed in the N:P ratio. With respect to total dissolved nitrogen, it was found that both nitrogen-amended treatments were significantly different but the phosphorus-amended treatment was not. This supports that our observations were not due to an isolation bias.

Based on the 16S rRNA sequences from the cultivated strains, we found that two different groups of gamma-proteobacteria responded to increases of nutrient availability in water and sediment environments, *Pseudomonas* and *Halomonas*, respectively. The strains characterized differed among the different treatments in the physiological characteristics analyzed, biofilm formation and antibiotic resistance. In contrast, genera such as *Shewanella, Citrobacter*, and *Rheinheimera* in water, as well as *Stenotrophomonas* and *Brachybacterium* in sediment were not isolated from the fertilized mesocosms, although they were present at time zero. Moreover, some rare genera, such as *Kocuria*, which was not documented at time zero, was found after the enrichment. However, we note that this rare genus of Actinobacteria has been obtained in past work at CCB (Cerritos et al., 2011), so it is not foreign to this environment. Several genera isolated in this study, had not been previously reported to grow in PIA medium (McCaig et al., 2001; Rajkowski and Rice, 2001; Falcone-Dias et al., 2012; Weiser et al., 2014).

All these changes in bacterial groups, which were isolated during different times in the experiment, suggest that an important community change at the bacterial level took place after the enrichment, however it should be taken carefully given the sampling method. It can be speculated that the original community in Lagunita was dominated by *K*-strategists, which rely on long-term survival on limited resources (Pianka, 1970; Fuchs et al., 2000; Singer et al., 2011). After the enrichment, the microbial community was dominated by faster-growing gamma-proteobacteria, which can be considered *r*-strategists, rapidly exploiting nutrient patches and then dying or becoming dormant after substrate exhaustion. Indeed, *Pseudomonas* has been previously characterized in general as a *r*-strategist, as pseudomonads rapidly colonize and grow on nutrient-rich environments (Juteau et al., 1999; Margesin et al., 2003), due to its metabolic versatility (Clarke, 1982; Hallsworth et al., 2003; Domínguez-Cuevas et al., 2006). This shift is important as it has been reported that *K*-strategists are expected to allocate more energy and interact in a broader way with their environment, for example developing strategies to cope with their environment, than to grow (Fontaine et al., 2003).

Although the experiment showed a reduction in diversity of the isolated strains and interactions as a whole, details of these responses of strains to the individual treatments were largely idiosyncratic and seemed independent of their phylum. For example, the group that includes strain AP29, affiliated with *Pseudomonas*, responded to the P treatment, while another group of *Pseudomonas*, which includes strain ENNP10, responded to the NNP treatment. However, another *Pseudomonas* group was abundant at the beginning of the experiment and remained abundant in the control, as well as in the P treatment (group including strains AP9, AC10, and 1C11) (Figure 1). The sudden availability of P in P-deficient environment could favor the dominance of the *Pseudomonas* genus, which has been reported to solubilize phosphate (Park et al., 2009; Parani and Saha, 2012). In sediment samples it was observed in both N+P treatments that *Halomonas* exhibited the greatest response. This observation is consistent with the fact that the *Halomonas* genus has been identified as capable of denitrification and may have taken advantage of the added NO_3_ in the NP and NNP treatments (Mormille et al., 1999; Guo et al., 2013).

The production of chemical compounds as bacteriocins and/or antibiotics is a common mechanism of antagonism among microorganisms (Riley and Gordon, 1999; Lenski and Riley, 2002; Riley and Wertz, 2002; Kirkup and Riley, 2004; Hibbing et al., 2010; Kohanski et al., 2010; Majeed et al., 2011, 2013; Pérez-Gutiérrez et al., 2013; Aguirre-von-Wobeser et al., 2014). In this study, we observed a general decrease in the antibiotic resistance in both the water and sediment environment, for all antibiotics except Carbenicillin, after the experiment (Table 3). This decrease likely reflects modifications in microbial survival strategies under different conditions, including the control were wind movement of both water and sediment, was restricted by the mesocosm tubes. Given the antagonistic network previously documented among *Pseudomonas* bacterial isolates from CCB (Aguirre-von-Wobeser et al., 2014), this antagonism could be due to the competition for resources. Following “microbial market logic” (Werner et al., 2014), without the acute nutrient limitation, the cost of producing antibiotics to repel competitors for a scarce resource is no longer beneficial in the case of increased P and the ideal N:P ratio, while in the NNP treatment, the community is so perturbed by the further limitation of P in relation to N that antagonism or cooperation through biofilm formation is no longer an economic option. On the other hand, in a rock—paper—scissors (RPS) model behavior scenario, that can be applied only for structured environment (Kirkup and Riley, 2004; Nahum et al., 2011) such as sediment or biofilm, the strains that produce toxins (*C*) kill sensitive strains (*S*), which outcompete resistant strains (*R*), which in turn outcompete C (Czarán et al., 2002; Kirkup and Riley, 2004). In this RPS game, the resistant and producer strains spend resources to keep the resistance, which in an enriched environment may be no longer needed, thus those strains are outcompeted by the sensitive strains (Kerr et al., 2002). Interestingly, this shift from a collection of isolates with a high prevalence of resistance to several antibiotic tested, to a collection dominated by sensitive strains was observed in both habitats: water and sediment (Figure 2), suggesting that the fact of predicting the neighborhood, as required for the rock-paper-scissor model, is not a requisite for antagonism, at least in the analyzed system.

Market logic suggests that local environments determine trade connections (Werner et al., 2014), and biofilm formation is a way to ensure a local environment both in water and sediment. The mechanisms of bacterial biofilm formation are processes by which single cells coordinate and implement the formation of complex surface-attached communities (Davey and O'Toole, 2000). Bacteria that reside within the biofilm are to some extent isolated from environmental stresses, such as desiccation or nutrient limitation (Danhorn et al., 2004). Complex interactions, in particular mutualistic behaviors, are expected in a biofilm, since the secretion of the matrix that forms the biofilm is a form of public good that will increase the survival of the coexisting partners and will avoid the presence of cheaters (Werner et al., 2014).

Biofilm-forming strains were found in our experiment in both habitats at time zero and in most of the enrichment treatments, suggesting that this cooperation strategy is more ingrained in the whole CCB microbial community than antagonistic interactions promoted by antibiotics, where rare species cannot afford to pay its costs. Biofilm formation was not present only in the sediments of the NNP treatment where P was further limited in relation to N. This treatment was dominated by strain of *Halomonas*. The NP treatment was also dominated by closely related strains of *Halomonas* that were biofilm-forming (Supplementary Figure 2). This differential response suggests that a wide range of strategies to cope with environmental limitations is present in CCB microbes, even within a single genus.

As the Black Queen Hypothesis (BQH) suggests, certain biological functions are not only expensive, but are also broadly distributed in the community, since they are public goods. Hence, the majority of the community can afford to lose those functions if they are at least a proportion of helpers that produce such public good (Morris et al., 2012). As a consequence, for a microbial market, we also need “price” differences and supply, as well as “demand” variation. In this study, we observed a shift in the composition of the bacterial community, from a diverse community prior the enrichment to a community dominated by few genera such as *Pseudomonas* in water or *Halomonas* in sediment after the addition of N+P. As previously stated, the change was not only related to the community composition, but also to its physiological characteristics as antibiotic resistances and the ability of biofilm formation. These changes could be explained with this microbial market theory as the supplies changed with the experiment the balance between cost and benefit.

According to the market theory, the benefit of trade depends not only on the interacting partners but also on the available supply of commodities from other sources and it is expected that biotic or abiotic conditions influence the demand for a particular service (Werner et al., 2014). Based on an analysis of Illumina tags of 16S rRNA (Elser et al., 2014, in preparation), our study site exhibits a large bacterial diversity dominated by alpha-proteobacteria and bacteroidetes. In the original community, we expected to find a strong competition among the members of this community. Under the original condition of extreme low P and N availabilities, it is predicted that specialization will be favored. All this considering, it is not surprising that when this particularly diverse and fragile network of interactions was perturbed by the mesocosm conditions and the nutrient input, the community structure and its biological market equilibrium changed, reducing its overall diversity, not only in the few cultured genera that we could follow, but in the overall community (Elser et al., 2014), suggesting that the resilience of this extremely oligotrophic oasis depends precisely on the permanence of such unbalanced stoichiometry.

### Conflict of interest statement

The authors declare that the research was conducted in the absence of any commercial or financial relationships that could be construed as a potential conflict of interest.

## Footnotes

^1^DifcoTM and BBLTM Manual, 2nd Edition. Pseudomonas Isolation Agar.
