# Supplementary material for: Enrichment experiment changes microbial interactions in an ultra-oligotrophic environment
Source: Front Microbiol. 2015 Apr 1;6:246. doi: 10.3389/fmicb.2015.00246 (PMC4381637; doi:10.3389/fmicb.2015.00246)
Supplement: Supplementary file 2 [file DataSheet2.DOCX]

***Supplementary Material***

**Enrichment experiment reduce diversity and changes microbial interactions in an ultra-oligotrophic environment**

**Gabriel Yaxal Ponce-Soto^1^, Eneas Aguirre-von-Wobeser^2^, Luis E. Eguiarte^1^, James J. Elser^3^, Zarraz M.-P. Lee^3^, Valeria Souza^1^***

^1^Laboratorio de Ecología Molecular y Experimental, Instituto de Ecología, Departamento de Ecología Evolutiva, Universidad Nacional Autónoma de México, México

^2^Red de Estudios Moleculares Avanzados, Instituto de Ecología A.C., México

^3^School of Life Sciences, Arizona State University, Arizona, USA

*** Correspondence:** Valeria Souza, Laboratorio de Ecología Molecular y Experimental, Instituto de Ecología, Departamento de Ecología Evolutiva, Universidad Nacional Autónoma de México, AP 70-275, Coyoacán, DF, 04510, México

souza@unam.mx

1. **Supplementary Figures**

**Supplementary Figure 1 | Antibiotic resistance and biofilm production in water isolates.** Antibiotic resistance and biofilm production assays from 863 water isolates, where each line represents one isolate. Each box, from left to right, represents the taxonomic affiliation of all the isolates and its corresponding antibiotic resistance and biofilm formation ability, respectively. The antibiotics tested were carbenicillin (Car), kanamycin (Kan), tetracycline (Tet), streptomycin (Str) and gentamycin (Gen).

**Supplementary Figure 2 | Antibiotic resistance and biofilm production in sediment isolates.** Antibiotic resistance and biofilm formation assays from 125 sediment isolates, where each line represents one isolate. Each box, from left to right, represents the taxonomic affiliation of all the isolates and its corresponding antibiotic resistance and biofilm formation ability, respectively. The antibiotics tested were carbenicillin (Car), kanamycin (Kan), tetracycline (Tet), streptomycin (Str) and gentamycin (Gen).
