# Supplementary figures and images for: Enrichment experiment changes microbial interactions in an ultra-oligotrophic environment
Source: Front Microbiol. 2015 Apr 1;6:246. doi: 10.3389/fmicb.2015.00246 (PMC4381637; doi:10.3389/fmicb.2015.00246)

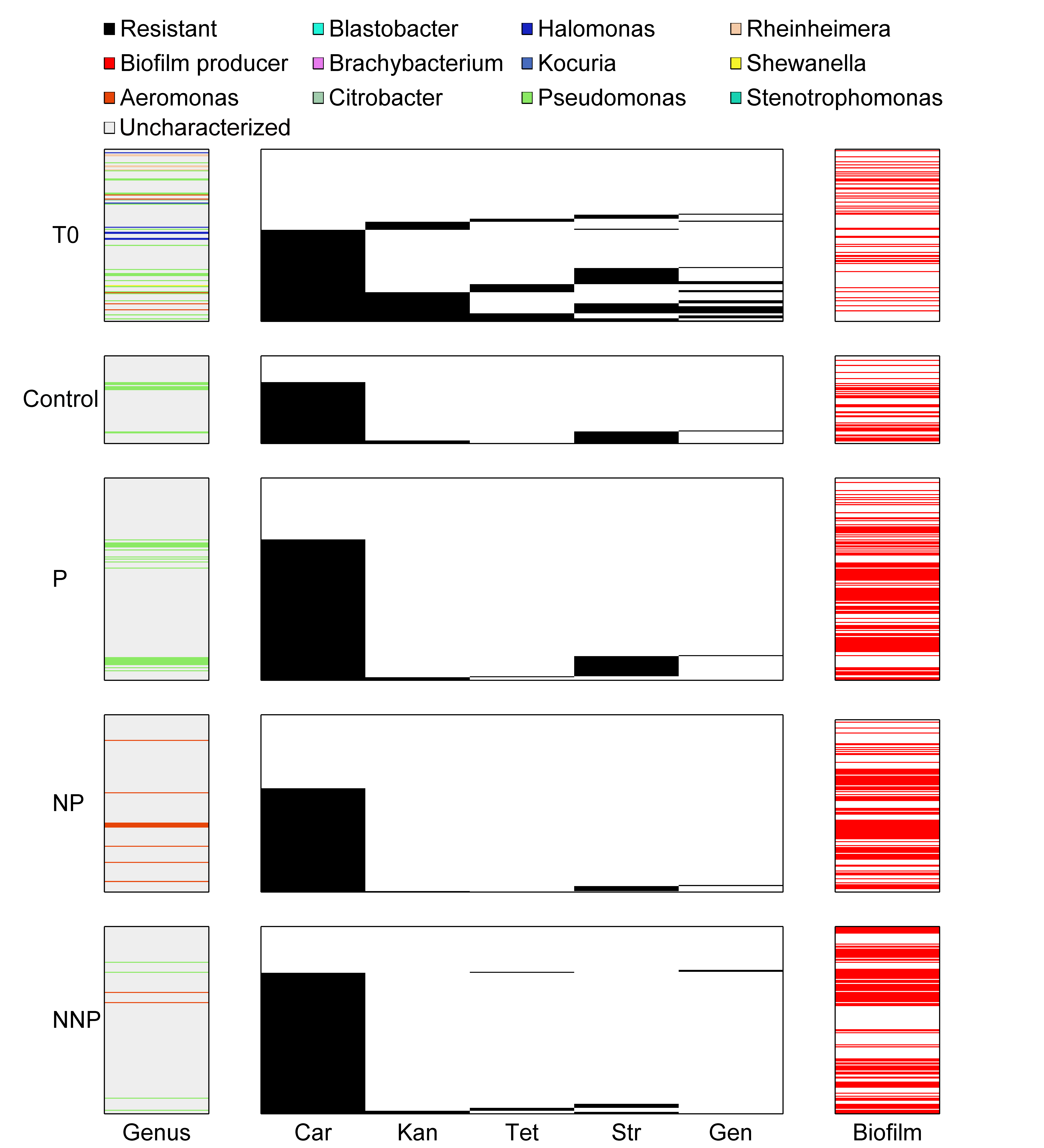

Supplement: Supplementary file 3 [file Image1.TIF]

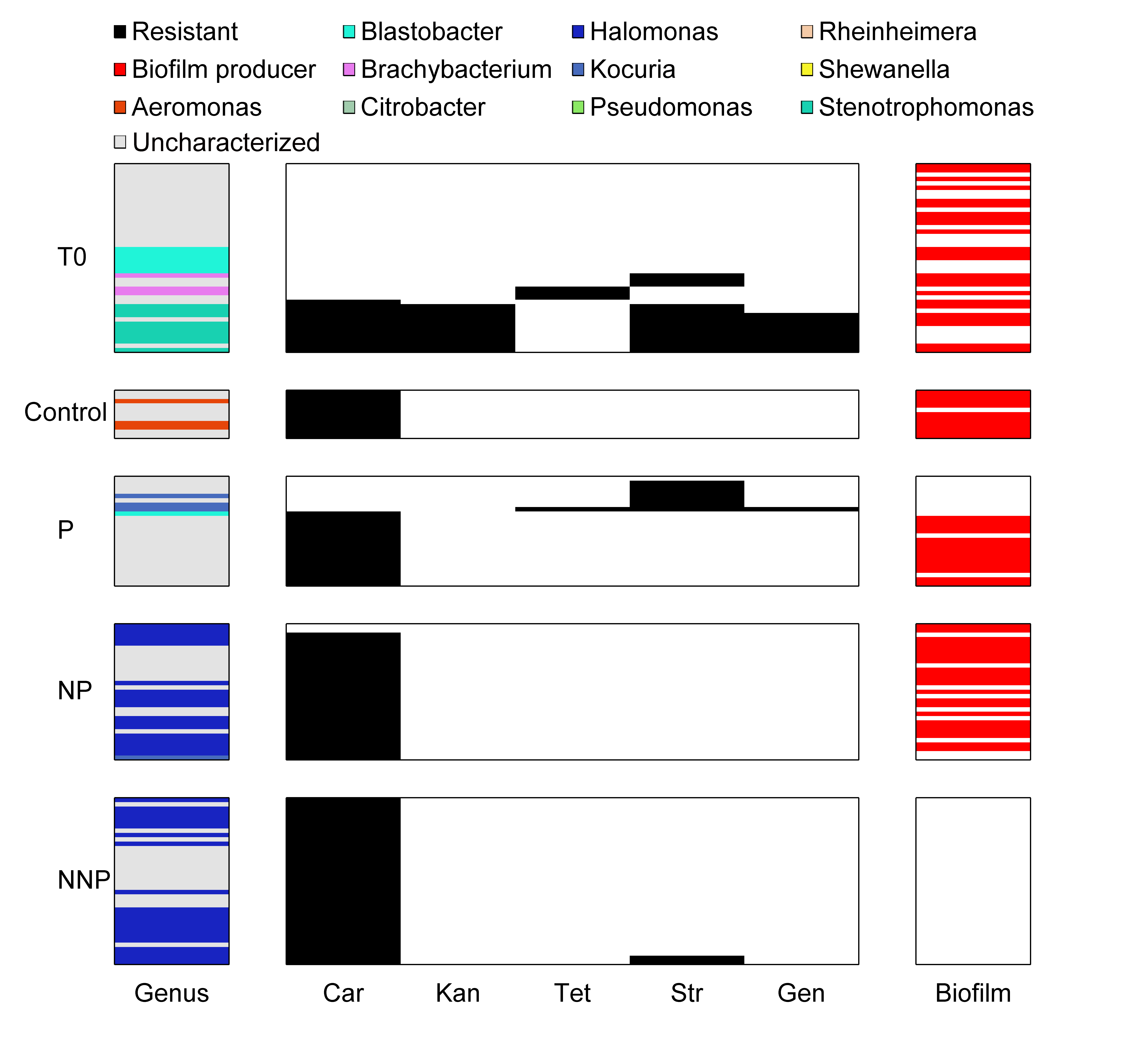

Supplement: Supplementary file 4 [file Image2.TIF]
